# Elevated Cardiac Troponin Is Most Often Associated With Type 2 Myocardial Infarction in Trauma Patients

**DOI:** 10.7759/cureus.39711

**Published:** 2023-05-30

**Authors:** John T Culhane, Jillian Drogan, Raymond I Okeke, Kishore Harjai

**Affiliations:** 1 Surgery, Saint Louis University School of Medicine, Saint Louis, USA; 2 Trauma Surgery, Saint Louis University School of Medicine, Saint Louis, USA; 3 General Surgery, SSM (Sisters of St. Mary) Health Saint Louis University Hospital, Saint Louis, USA; 4 Cardiology, Saint Louis University School of Medicine, Saint Louis, USA

**Keywords:** revascularization, cardiology evaluation, myocardial infarction, troponin, trauma

## Abstract

Introduction

Cardiac troponin (cTn) forms an essential part of the diagnostic criteria for myocardial infarction (MI). Type 1 MI is a primary coronary arterial event, whereas type 2 MI is due to coronary oxygen supply/demand mismatch, which is common in trauma patients. In addition, cTn may be elevated for many reasons other than MI. cTn elevations in trauma may not be specific for MI amenable to revascularization. The aim of this study is to determine which subset of trauma patients benefits from measuring cTn, and which patients with elevated cTn benefit from ischemic workup.

Methods

This is a retrospective cohort study. All patients on the trauma service of a level 1 trauma center with cTn elevated above the upper reference value of 0.032 ng/ml from July 2017 through December 2020 were selected. Baseline characteristics were recorded. The main outcomes were cardiology determination of the etiology of elevated cTn and patient survival. Logistic regression was used for multivariate analysis.

Results

One hundred forty-seven (147; 1.1%) of 13746 trauma patients had maximum cTn over the 99th percentile. Forty-one (27.5%) of the 147 had ischemic changes on electrocardiogram (ECG). Sixty-four (43.0%) had chest pain. In 81 (55.1%) cases, cTn was ordered without a clearly justified indication. One hundred thirty-seven patients (93.3%) received a cardiology consult. Two (1.5%) of 137 patients had a type 1 MI, which was diagnosed by ECG and clinical symptoms before cTn results were available. One hundred thirty-five patients were evaluated for cardiac ischemia based on elevated cTn. In 91 (66.4%) cases, the elevated cTn was attributed to a cardiac oxygen supply/demand mismatch. The etiology was cardiac contusion for 26 (19.0%), with the rest attributed to various other trauma-related causes. The cardiology consult changed management for 90 (65.7%) patients, mainly consisting of further evaluation by echocardiogram for 78 (57.0%) patients. Elevated cTn was a significant independent predictor of death with an adjusted odds ratio of 2.6 (p=0.002).

Conclusion

Isolated cTn values in trauma are most often due to type 2 MI resulting from trauma-related issues, such as tachycardia and anemia, which affect myocardial oxygen supply and demand. Changes in management generally consisted of further workup and interventions such as monitoring and pharmacologic treatment. Elevated cTn in this cohort never led to revascularization but was valuable to identify patients who required more intensive monitoring, longer-term follow-up, and supportive cardiac care. More selective ordering of cTn would improve specificity for patients requiring specialized cardiac care.

## Introduction

Cardiac troponin (cTn) forms an essential part of the diagnostic criteria for myocardial infarction (MI). Cardiac troponin elevated beyond the 99th percentile is a necessary condition to meet the universal definition of myocardial infarction (MI) along with typical clinical signs and electrocardiogram (ECG)/echocardiogram findings [1]. Cardiac troponin is ordered frequently in many contexts, with elevated values generally prompting a cardiac evaluation [2-4]. Elevated cTn, however, is not specific for MI with even lower specificity for type 1 MI [5].

Elevated cTn is frequently due to trauma-related conditions such as cardiac contusion, hyperadrenergic state, hypovolemic shock, tachycardia, or systemic inflammation due to injury. Concomitant non-trauma conditions, such as congestive heart failure, arrhythmia, myocarditis, pulmonary embolism, renal failure, or sepsis, may also contribute to elevated cTn in trauma patients [6]. The outcome of type 1 MI may be improved with evidence-based treatment [7]. Concern for missing MI and desire for efficient rule-out algorithms has resulted in a sacrifice of specificity in favor of sensitivity. Recently, the problem has been further exacerbated by the introduction of high-sensitivity cTn [8]. In practice, few trauma patients with elevated cTn have type 1 MI. In most cases, the high cTn is attributed to a supply-demand mismatch. While this does not generally require revascularization, it still may benefit from the involvement of specialists to stratify risk and manage sequelae such as arrhythmia [9].

While cardiac evaluation and supportive care are useful for both types 1 and 2 MI, elevated cTn does not change management in every patient. Overuse and inappropriate interpretation of cTn can lead to a waste of resources and a risk of overdiagnosis [10]. The trauma population is especially vulnerable to the overinterpretation of elevated cTn due to the increased likelihood of other explanations for the abnormality. This raises the question of what we can do to improve the specificity of cTn in trauma. The aim of this study is to determine which subset of trauma patients benefits from measuring cTn and which patients with elevated cTn benefit from ischemic workup. Our hypothesis is that more selective ordering of cTn in the appropriate clinical context can improve specificity without missing conditions that require cardiac intervention.

## Materials and methods

Study design

This is a retrospective cohort study of patients admitted to the trauma service of a level one trauma center.

Data collection

All trauma admissions for the years July 2017 through December 2020 were reviewed for elevated cTn values. Patients were selected from a prospectively maintained registry. Direct chart review was conducted to review the cardiology assessment and detailed outcomes of all the patients. Cardiology consultation notes, follow-up notes, and results of cardiac testing were reviewed to determine the cardiology assessment of the etiology of elevated cTn. Clinical notes were reviewed to determine patient outcomes and whether mortality was cardiac-related.

Inclusion and exclusion criteria

The cTn used in our hospital was troponin I, with a reference range of <0.032 ng/ml. All patients admitted to the trauma service with at least one measured cTn above the 99th percentile reference value were selected. We used the reference range for the specific assay reported for each patient, which showed slight variation over time. A chart review was performed to identify any patients with chronically elevated cTn. In the absence of documented chronic elevation, elevated cTn levels were assumed to be acute. cTn was followed temporally to confirm the downtrending values. Patients were selected for acute change in cTn versus chronic elevation. Exclusions were: transfer from an outside facility with known MI, cardiac arrest in the field, and penetrating cardiac trauma. All patients received definitive care in our tertiary care hospital.

Statistical analysis

Descriptive data were listed as the number of patients in each category with the corresponding percentage of the elevated cTn cohort. Multivariate analysis was performed with multiple logistic regression. The outcome was death. Predictors in the model were age, sex, blunt mechanism, body mass index (BMI), Glasgow Coma Scale (GCS), injury severity score (ISS), trauma-related injury severity score (TRISS), systolic blood pressure (SBP), and elevated cTn.

## Results

One-hundred forty-seven (147; 1.1%) of 13746 trauma patients had maximum cTn over the 99th percentile. Ninety-three (63.3%) were male. The mean age of the cohort was 68.7 years. Forty-six (31.3%) had a previous diagnosis of coronary artery disease. One hundred thirty-seven (93.3%) received a cardiology consult. For all patients, the maximum cTn ranges from 0.03 to 164.86 ng/mL. The range of maximum cTn for type 1 MI was 16.32 to 39.46 ng/ml. The lowest value in the range is 510.0 times the upper reference value.

Table 1 displays the baseline demographic and physiologic characteristics of the patients, along with characteristics of the traumatic injury. ISS is included as an anatomic injury scale and TRISS as a physiologic scale. The number of units of blood is included as an additional surrogate for injury severity.

**Table 1:** Baseline Characteristics standard deviation (SD), injury severity score (ISS), trauma-related injury severity score (TRISS), systolic blood pressure (SBP), cardiac troponin (cTn)

Characteristic (n, %)	No elevated cTn	Elevated cTn
Male sex	9250 (68.0%)	93 (63.3%)
Blunt mechanism	10712 (63.4%)	124 (86.7%)
Characteristic (mean, SD)		
Age	47.5 (20.8)	69.2 (17.3)
Body mass index	28.5 (16.4)	28.1 (8.0)
Glasgow Coma Scale	13.8 (3.2)	12.9 (4.0)
ISS	11.9 (10.4)	17.5 (13.0)
TRISS	0.92 (0.20)	0.84 (0.25)
Systolic blood pressure	134.7 (31.0)	136.8 (33.9)
Pulse	87.2 (22.5)	89.5 (21.0)
Units of blood transfused	1.68 (1.10)	1.71 (1.20)

Table 2 displays the reasons that the provider ordered the cTn. If a detailed review of the notes did not reveal any reason, the category of “no documented indication” was assigned. The summary of indications section of the table categorizes indications as non-selective if the reason for ordering the cTn is not provided or if the cTn was ordered due to a fall clearly described as mechanical rather than provoked by cardiac syncope. The indications are designated as questionably selective if the indication is poorly specific for cardiac ischemia.

**Table 2:** Reason for Ordering cTn Documented reason for ordering cardiac troponin (cTn). Numbers may differ from the total number of each category in the population if the condition was not the documented reason for ordering cTn.

Indications for ordering cTn	Frequency	Percentage (%)
Non-specific ECG	35	23.8
Chest trauma	28	19.0
No documented indication	22	15.0
Mechanical fall	16	10.9
Chest pain	12	8.2
ECG suspicious for ischemia (STEMI, ST elevation, new ST depression, new left bundle branch block)	12	8.2
Syncopal fall	8	5.4
Hemodynamic instability	7	4.8
Respiratory symptoms	3	2.0
Cardiac arrest	2	1.4
Pulmonary embolism (PE)	1	0.7
Right heart failure	1	0.7
Summary of Indications		
Non-selective (No documented indication or mechanical fall)	37	25.2
Questionably selective (Non-selective + non-specific ECG + syncopal fall + PE)	81	55.1

Table 3 displays the ECG findings of the patients with elevated cTn.

**Table 3:** ECG Findings With Elevated cTn Ischemia includes final ECG interpretations including STEMI, infarct, and ischemia. Right bundle branch block (RBBB), left bundle branch block (LBBB), atrioventricular (AV), cardiac troponin (cTn), ST-elevation myocardial infarction (STEMI) The total exceeds 147 due to the possibility of more than one ECG abnormality per patient.

ECG findings with elevated cTn	Frequency	Percentage (%)
Non-specific ST changes	42	28.6
Ischemia	41	27.9
Atrial fibrillation	27	18.4
RBBB	17	11.6
AV conduction abnormality	16	10.9
Normal	13	8.8
LBBB	10	6.8
STEMI	1	0.7
Other or not recorded	35	27.9

Table 4 displays the conclusion of the consulting cardiologists regarding the etiology of the elevated cTn. The conclusions were derived from the initial consultation and follow-up notes and testing. A Shrock shunt is the emergent surgical bypass of an injured retro hepatic inferior vena cava.

**Table 4:** Etiology of Elevated cTn Determined by Cardiology The total exceeds the 137 patients evaluated by cardiology because each patient may have more than one factor contributing to elevated cardiac troponin (cTn).

Etiology of elevated cTn	Frequency	Percentage (%)
Demand ischemia/Type 2 MI	91	66.4
Contusion/direct trauma	26	19.0
Traumatic brain injury	9	6.6
Atrial fibrillation	4	2.9
Type 1 MI	2	1.5
Aortic stenosis	2	1.5
Acute kidney injury	2	1.5
Heart failure exacerbation	2	1.5
Myopericarditis	2	1.5
Takotsubo cardiomyopathy	1	0.7
Right ventricular strain	1	0.7
IVC injury with Shrock shunt	1	0.7
Pacemaker-induced tachycardia	1	0.7

One patient with type 1 MI had minor trauma and classic symptoms of chest pain with ST depressions on ECG. He was treated with a coronary artery bypass graft (CABG). The other had chest pain with ST-elevation myocardial infarction (STEMI) diagnosed on ECG. He was treated with percutaneous coronary intervention (PCI). These were the only two patients treated with coronary revascularization. After excluding these two patients because the initial diagnosis was not based on cTn, all remaining MIs were type 2. The positive predictive value of cTn for type 1 MI in this dataset is zero.

Table 5 displays the changes in management prompted by the cardiology consultation for the elevated cTn. Most changes in management consisted of further testing by echocardiogram, but a few patients underwent more invasive testing. A few patients were treated pharmacologically with agents such as beta blockers and aspirin.

**Table 5:** Intervention for Elevated cTn Changes in management prompted by the elevated cardiac troponin (cTn) in patients evaluated by cardiology. Patients with type 1 MI diagnosed prior to cTn testing and those not evaluated by cardiology are excluded (n=135). Neither patient undergoing cardiac catheterization required therapeutic intervention.

Intervention for elevated cTn	Frequency	Percentage (%)
Echocardiogram	78	57.8
No change in management	47	34.8
Pharmacologic management	8	5.9
Stress Test	2	1.5
Cardiac Catheterization	2	1.5

Table 6 displays the outcome of the patients with elevated cTn specifically with respect to cardiac-related mortality.

**Table 6:** Patient Outcomes Patients with type 1 MI diagnosed prior to Cardiac troponin (cTn) testing are excluded (n=145). A cardiac event is defined as an event other than a supply/demand mismatch.

Patient Outcomes	Frequency	Percentage (%)
No death or cardiac event	115	79.3
Death from an unknown cause	13	9.0
Death from a non-cardiac cause	12	8.3
Definite a cardiac-related death	3	2.1
Possible a cardiac-related death	2	1.4

Table 7 shows the results of a multivariate logistic regression. The outcome is mortality. The dependent variable of interest is elevated cTn. Various demographic and trauma-related covariates are included to control for potential confounding variables. 

**Table 7:** Multivariate Predictors of Death body mass index (BMI), Glasgow Coma Scale (GCS), injury severity score (ISS), trauma-related injury severity score (TRISS), systolic blood pressure (SBP) SE (coef) is the standard error of the coefficient representing the expected change in log odds associated with a unit change in each predictor variable.

Variable	OR	SE (coef)	p	CI of OR
Age	1.05	0.004	<0.001	(1.044-1.060)
Sex	0.78	0.142	0.082	(0.592-1.032)
Blunt trauma	0.49	0.192	<0.001	(0.338-0.717)
BMI	0.99	0.005	0.797	(0.988-1.009)
GCS	0.77	0.028	<0.001	(0.731-0.815)
ISS	1.08	0.007	<0.001	(1.067-1.095)
TRISS	1.30	0.494	0.598	(0.493-3.414)
SBP	1.01	0.002	0.003	(1.002-1.009)
Elevated cTn	2.60	0.307	0.002	(1.427-4.752)

## Discussion

Troponin forms a component of the contractile structure of muscle tissue, mediating the interaction between actin and myosin. Troponins T and I are known as cardiac troponins (cTn) due to their specificity for myocardial tissue [5]. Troponin released by injured myocardium is now recognized as the most accurate biomarker of myocardial infarction. At least one cTn value above the 99th percentile upper reference limit is diagnostic of myocardial injury but not necessarily myocardial infarction. According to the universal definition of myocardial infarction, elevated cTn must be accompanied by at least one of the following: symptoms of myocardial ischemia, characteristic ECG changes, and/or imaging evidence of new loss of viable myocardium or wall motion abnormality [1].

An important goal of an ischemic cardiac workup is early identification and treatment of type 1 MI, which is due to acute plaque rupture within coronary arteries. Type 1 MI benefits from time-dependent evidence-based therapy. Prompt antithrombotic and antiplatelet medication and revascularization are known to improve outcomes [1]. Although cTn is essential in the workup of MI, it is not specific for type 1 MI. Furthermore, type 1 MI is generally identified by ECG changes and clinical symptoms with cTn serving as confirmation. Myocardial necrosis due to oxygen supply/demand mismatch constitutes type 2 MI. Generally, treatment of type 2 MI consists of correcting the underlying condition that creates the mismatch [11]. Correcting physiologic derangements is a standard therapeutic priority, however, the diagnosis of type 2 MI serves to alert the provider that the condition is severe enough to damage the myocardium. This should lead to greater therapeutic urgency to assess damaged myocardium and to correct the underlying condition.

Elevated cTn is not specific to MI; in fact, elevated cTn is common. In a study of sequential emergency department (ED) patients, Sandoval et al found that one-third of non-selected patients had elevated cTn [12]. Carroll et al. found that 40% of patients in a trauma ICU had elevated cTn. The cause was generally global stress on the heart rather than MI [13]. Recently, high-sensitivity cTn has further increased the number of positive results. Fifth-generation assays can detect cTn at 10-to-100-fold lower concentrations than earlier tests. While this improves the negative predictive value, making the test useful to rule out MI, it also makes the interpretation of positive results more difficult [14]. This greater sensitivity does not always benefit the patient. More sensitive cTn assays have doubled the diagnoses of type 2 MI without improved outcomes [8].

The first step in reducing false positive results is appropriate testing. In general, the proper indications for a cTn assay are chest pain or other possible ischemic symptoms, and ischemic changes on ECG. A program designed to limit the use of this indication resulted in less testing, with no worsening of outcomes [3]. However, cTn is often ordered non-selectively. A recent study of a United States (US) emergency department (ED) showed that cTn was measured in 9109 (19%) patients. Seventy-seven percent (77%) of these were classified as non-selective, meaning that there was no suspicion of MI [15]. When cTn is ordered indiscriminately as in the US, the positive predictive value (PPV) is low [4]. Cardiac troponin levels ordered in the ED are not always part of a well-thought-out diagnostic strategy, making them prone to overuse [2].

Cardiac troponin in trauma is appropriately used to work up signs and symptoms of cardiac ischemia, but there are a few additional indications specific to trauma. The 2012 Eastern Association for the Surgery of Trauma guidelines issued a level 2 recommendation to measure cTn any time a blunt cardiac injury (BCI) is suspected. If ECG and cTn are normal, BCI is ruled out. If cTn is abnormal, the guidelines recommend monitoring for arrhythmia. Further workup is reserved for hemodynamic instability or persistent arrhythmia [16]. A 2020 study confirmed that high-sensitivity cTn predicted poor outcomes in BCI [17]. Thus, measuring cTn in BCI is considered the standard of care, specifically to determine who requires monitoring for arrhythmia, not for predicting MI.

In trauma, there is abundant evidence of the prognostic value of cTn independent of cardiac diagnosis. Kalbitz et al. found that elevated cTn correlated with ISS, pressor requirements, and survival [18]. Hammarsten et al. reported that cTn levels are linked to poor prognosis in trauma regardless of cause [6]. Elevated cTn even has prognostic value in pediatric trauma patients [19]. Cardiac troponin may rise due to non-ischemic causes such as the physiologic stress of exercise [6]. In a group of 1081 trauma ICU patients, Martin et al. found that cTn elevation in trauma correlated more closely with the overall injury severity and physiologic stress than cardiac pathology [20]. Blunt cardiac injury may cause direct release of cTn [16,18]. Traumatic brain injury is also an independent predictor of elevated cTn. This may be due to a catecholamine surge associated with stress and inflammation [21]. Changes in supply and demand are common. Anemia, hypoxia, and hypotension restrict myocardial oxygen supply. Tachycardia, hyperadrenergic state, and systemic inflammation increase metabolic rate and cause the heart to consume more oxygen [20].

Over-testing and overdiagnosis of MI involve serious risks. The diagnosis of MI can lead to the use of anticoagulation and antiplatelet agents in patients prone to bleeding. The stigma of MI may result in a reluctance to perform surgical procedures. In order to reduce the risk of false positive cTn results, we propose the following algorithm for cardiac biomarker use and interpretation in trauma:

Order cTn when there is a genuine suspicion of MI, not just for old age, mechanical falls, or other non-specific indications. Perform ischemic workup for suspicion of MI consistent with the universal definition. This requires not only elevated cTn but also ischemic symptoms not attributable to the traumatic injury or ischemic changes on ECG. When symptoms are not clear or ischemic changes are non-specific, cardiology consultation is valuable to help guide the workup. If the patient has a cardiac contusion, monitor for arrhythmia but order cTn and conduct further cardiac ischemic workup only for persistent arrhythmia or abnormal hemodynamics. If the cardiac supply/demand mismatch explains the elevated cTn, monitor the patient while addressing the source of the imbalance.

Limitations

Our study relies on a single institution dataset with a low rate of positive outcomes. We do not have enough cases to both develop and validate a complex model. Due to the low incidence of MI in our cohort, we could have chosen many thresholds with a perfect discriminating ability, but these are subject to type 2 errors. The magnitude of cTn change may have influenced the diagnosis of MI, introducing an ascertainment bias. A large registry is ideal to study a rare outcome but existing registries, such as the National Trauma Data Bank, lack sufficient granularity to investigate the performance of individual laboratory tests.

## Conclusions

Most trauma patients with elevated cardiac troponin (cTn) were ultimately diagnosed with type 2 myocardial infarction (MI). In the trauma population, isolated cTn values have a poor positive predictive value (PPV) for type 1 MI. No patient requiring revascularization was identified by a cTn result; however, cardiology evaluation leads to benefits other than revascularization. Most elevated cTn results changed management. Most patients underwent further workup and a clinically important number had changes to their therapy.

There is still room for improvement of specificity for cTn in trauma. Many patients had cTn ordered with no objective criteria to suspect MI. Trauma patients have many alternative diagnoses to explain elevated cTn. Ordering cTn more selectively and evaluating cTn in the context of the injury pattern and severity is necessary to improve the PPV of laboratory cardiac testing.
